# *Leishmania tarentolae*: Taxonomic classification and its application as a promising biotechnological expression host

**DOI:** 10.1371/journal.pntd.0007424

**Published:** 2019-07-25

**Authors:** Stephan Klatt, Larry Simpson, Dmitri A. Maslov, Zoltán Konthur

**Affiliations:** 1 Max Planck Institute of Colloids and Interfaces, Potsdam, Germany; 2 Department of Microbiology, Immunology and Molecular Genetics, Geffen School of Medicine at UCLA, University of California, Los Angeles, California, United States of America; 3 Department of Molecular, Cell, and Systems Biology, University of California, Riverside, California, United States of America; Institut Pasteur de Tunis, TUNISIA

## Abstract

In this review, we summarize the current knowledge concerning the eukaryotic protozoan parasite *Leishmania tarentolae*, with a main focus on its potential for biotechnological applications. We will also discuss the genus, subgenus, and species-level classification of this parasite, its life cycle and geographical distribution, and similarities and differences to human-pathogenic species, as these aspects are relevant for the evaluation of biosafety aspects of *L*. *tarentolae* as host for recombinant DNA/protein applications. Studies indicate that strain LEM-125 but not strain TARII/UC of *L*. *tarentolae* might also be capable of infecting mammals, at least transiently. This could raise the question of whether the current biosafety level of this strain should be reevaluated. In addition, we will summarize the current state of biotechnological research involving *L*. *tarentolae* and explain why this eukaryotic parasite is an advantageous and promising human recombinant protein expression host. This summary includes overall biotechnological applications, insights into its protein expression machinery (especially on glycoprotein and antibody fragment expression), available expression vectors, cell culture conditions, and its potential as an immunotherapy agent for human leishmaniasis treatment. Furthermore, we will highlight useful online tools and, finally, discuss possible future applications such as the humanization of the glycosylation profile of *L*. *tarentolae* or the expression of mammalian recombinant proteins in amastigote-like cells of this species or in amastigotes of avirulent human-pathogenic *Leishmania* species.

## Author summary

Recent studies indicate that the classification of *Leishmania* species into one of the four currently recognized subgenera (*L*. [*Leishmania*], *L*. [*Sauroleishmania*], *L*. [*Viannia*], and *L*. [*Mundinia*]), or even the related genera, can be a challenging task. A monotonous morphology of this group is coupled with a high genetic variability in nature, a multitude of clinical manifestations, and a propensity for rapid evolution in culture. As some geographic regions have not been adequately sampled, the known biodiversity of this group may be an underestimate of its true dimensions. Therefore, the taxonomic classification of the genus *Leishmania* and its close relatives is not definitively settled. In this review, we will focus on biotechnological applications of *L*. *tarentolae*, a protozoan parasite of geckos. As this species belongs to the genus *Leishmania* and subgenus *L*. (*Sauroleishmania*), and because many Leishmania are human-pathogenic, the taxonomic status and phylogenetic position of *L*. *tarentolae* are relevant for biosafety and also discussed herein. The subgenus of *L*. (*Sauroleishmania*) mostly contains species that are infectious to reptiles, but some species and strains, such as *L*. *adleri* and *L*. *tarentolae* strain LEM-125, were also shown to be (transiently) infectious to humans. However, most strains of *L*. *tarentolae* are nonpathogenic to humans and can be easily handled as laboratory culture. Therefore, this species has been a successful model system for representing other Leishmania in basic research. It has also been of great interest for the scientific community, as it represents a promising host for the expression of human recombinant proteins (including glycoproteins and its future application for the expression of full-length antibodies) and immunotherapy agent for human leishmaniasis treatment. These two topics will also be discussed.

## Introduction

*L*. *tarentolae* is a eukaryotic protozoan parasite commonly regarded as not pathogenic to humans. It belongs to the genus *Leishmania*, which currently includes more than 50 species, and to the subgenus *Sauroleishmania* [1]. The fact that the 21 sauroleishmanial species form a monophyletic group that belongs to the genus *Leishmania* is firmly established. However, some members of the clade show phenotypic peculiarities indicating that the group is not homogenous. For example, *L*. *adleri*, unlike most sauroleishmanias, can infect mammals [2], and the two main *L*. *tarentolae* strains, TAR and LEM, show different behavior in cell culture and can be transiently infectious [3, 4]. Out of all sauroleishmanias, *L*. *tarentolae* is the best-studied saurian-pathogenic species today [5]. Initially, the parasite was primarily used as a model species to study kinetoplastid DNA (kDNA) organization, RNA editing [6–8], and gene amplification [9]. Recombinant protein production is another important application area. As most strains of this species are not pathogenic to humans, it is relatively easy and cost effective to cultivate, and it offers robust recombinant protein yields (0.1–30 mg/L) [10, 11]. Additionally, the expression system can easily be expanded to an industrial production scale. Furthermore, *L*. *tarentolae* is able to express functional mammalian antibody fragments and human glycoproteins [12–14], which is a common pitfall of many other heterologous expression systems (such as *Escherichia coli* or yeast) because of the lack of correct posttranslational protein modifications (PTMs), protein folding difficulties, and the occurrence of inclusion bodies. In the case of two human glycoproteins, *L*. *tarentolae* showed high PTM homogeneity to the human counterpart with complex biantennary N-glycans (erythropoietin [EPO] [15]) and initial O-glycans (soluble amyloid precursor protein alpha [sAPPalpha] [16]). In the case of the N-glycans, only terminal sialic acids (N-acetylneuraminic acids) were missing. In addition, the N-glycosylation pattern was shown to be exceptionally homogenous. Multisubunit proteins, like the human heterotrimeric glycoprotein laminin (LM)-322, were also functionally expressed in *L*. *tarentolae* [17]. Because full-length antibodies are multisubunit proteins and are also glycosylated, it is likely that they will also be functionally expressed in *L*. *tarentolae*. Another growing and promising application area of *L*. *tarentolae* is its use as a prophylactic vaccine as well as a therapeutic approach to treat human leishmaniasis. For example, immunization with recombinant *L*. *tarentolae* was shown to protect BALB/c mice against *L*. *infantum* infection. [18].

In this review, we will discuss the taxonomy and phylogeny of the genus *Leishmania*, including the position of sauroleishmanial species. We will further review the life cycle of *L*. *tarentolae* and its geographical distribution and highlight some differences and similarities to human-pathogenic *Leishmania* species. The current biosafety level of *L*. *tarentolae* is also discussed, with a focus on the TARII and LEM-125 strains. Furthermore, we will summarize the current state of biotechnological research (as introduced above) and explain in more detail why *L*. *tarentolae* is an advantageous and promising human recombinant protein expression host. We will outline possible future applications like the humanization of the glycosylation profile of *L*. *tarentola*e with the trans-sialidase (TS) gene and the expression of mammalian recombinant proteins in amastigote-like cells of *L*. *tarentolae* or in an avirulent strain (amastigotes) of human-pathogenic *Leishmania* species.

## Methods

To write this review, we conducted multiple searches on PubMed and Google Scholar with the following keywords (one, two, or more words combined; deadline of online search, March 2019): *Leishmania tarentolae*, taxonomy, phylogeny, genus classification, life cycle, geographical distribution, genome, kinetoplast/kinetoplastid, RNA editing, gene amplification, U insertion/deletion, protein expression, biotechnological application, glycoprotein, antibody, expression vector, culture conditions, LEXSY, TARII strain, UC strain, LEM strain, *Sauroleishmania*, *Leishmania adleri*, *Leishmania donovani*, amastigotes, promastigotes, sand flies, human pathogenic, vaccine candidate, antileishmanial drug, and immunotherapy agent. Suitable manuscripts and reviews were selected, and the information was summarized into the four main sections: (1) Taxonomic classification of *L*. *tarentolae*, (2) Biotechnological applications of *L*. *tarentolae*, (3) Useful online tools/databases, and (4) Discussion and outlook (to discuss and suggest potential future applications). In addition, most main sections include several subsections.

## Taxonomic classification of *L*. *tarentolae*

*L*. *tarentolae* belongs to the subgenus *Sauroleishmania*, which along with additional subgenera (*Leishmania*, *Viannia*, *Mundinia*) and the related organisms constitute the genus *Leishmania*. Although the taxonomic classification of the genus *Leishmania* is not definitively settled, the species of lizard parasites (*Sauroleishmania*) are regarded as a bona fide member of this group. Here, we will discuss the current taxonomic issues and give insights into the life cycle and geographical distribution of *L*. *tarentolae* and compare its genome with the genomes of human-pathogenic species.

### The genus *Leishmania*

The genus *Leishmania* belongs to the class Kinetoplastea and the order of Trypanosomatida [1, 19]. All kinetoplastids possess a DNA-containing region inside their single large mitochondrion, which is called the kinetoplast. Since its first description by W. B. Leishman [20], C. Donovan, and J. H. Wright [21] in 1903–1904, more than 50 *Leishmania* species, which infect mammals and reptiles and are transmitted by sand flies, are recognized today [1].

The classification of the genus *Leishmania* is still in flux, although the phylogenetic relationships among these organisms are becoming increasingly clear. These dixenous parasites of mammals and reptiles form a coherent group on the phylogenetic trees [22]. This group emerges relatively late from the monoxenous parasites of insects, and its monophyletic origin is well supported [19, 23, 24]. Two major phylogenetic lineages (referred to as sections) are recognized within this clade: Euleishmania and Paraleishmania [25]. Each lineage is further subdivided into a series of subclades representing the subgenera. Although the earlier phylogenies did not confidently reveal the exact branching order among the subclades, recent analyses utilizing genomic datasets have shown a more defined picture [22, 24].

**Fig 1:**
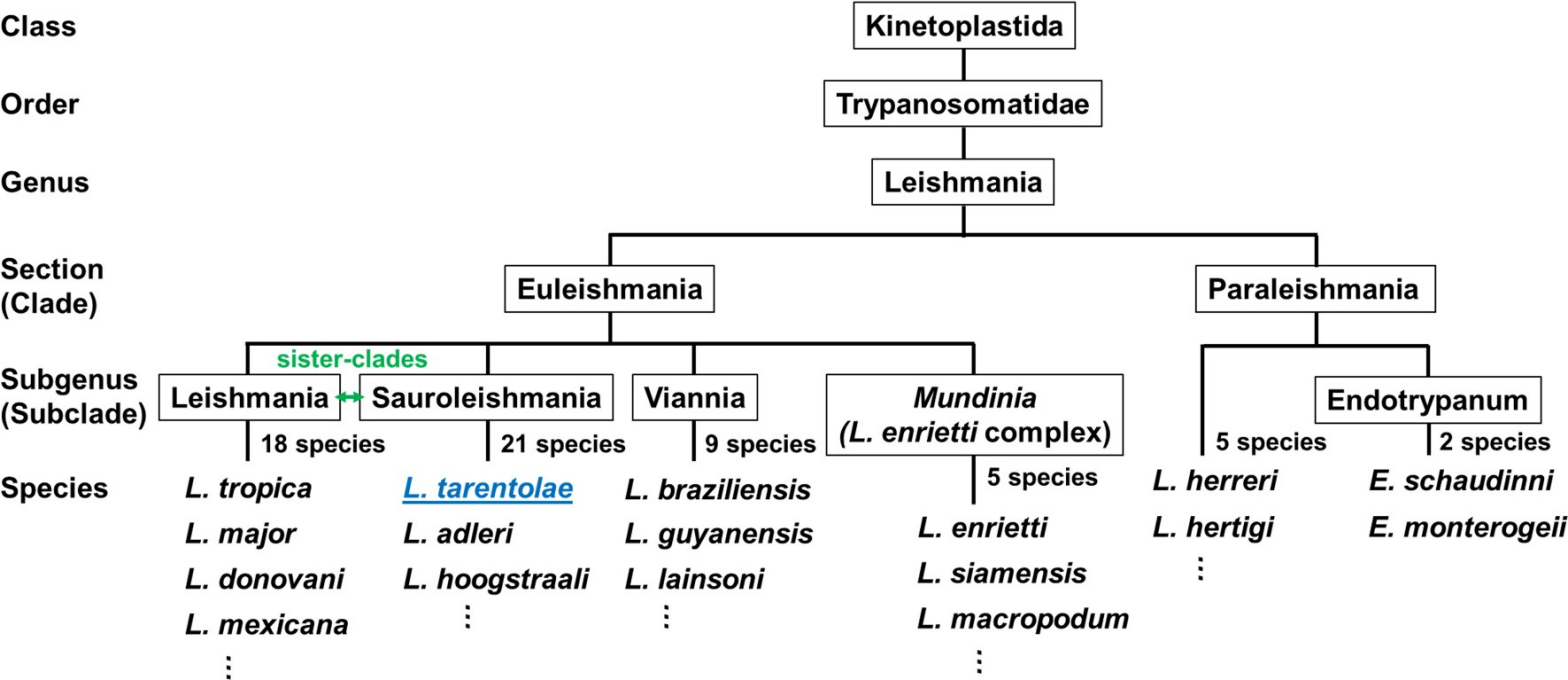
A phylogeny-based classification of the genus *Leishmania*. *L*. *tarentolae* belongs to the subgenus of *Sauroleishmania*, which is made up of mostly lizard-infecting parasites. *Adapted from [1]*.

There is no disagreement among taxonomists that the Euleishmania section represents the bona fide genus *Leishmania* and its subclades. The subgenera are as follows: *L*. (*Leishmania*), *L*. (*Sauroleishmania*), *L*. (*Viannia*), and *L*. (*Mundinia*), which is sometimes referred to as the "*L*. *enriettii* complex" [19, 24]. It is important to mention that the subgenera *L*. (*Leishmania*) and *L*. (*Sauroleishmania*) are sister-clades that diverged relatively late. Thus, the data unequivocally support the origin of lizard *Leishmania* from parasites of mammals. This phylogenetic position was the main argument for the inclusion of these reptilian parasites into the genus *Leishmania* as a subgenus rather than separating *Sauroleishmania* as a separate genus [26]. A simplified classification/phylogenetic tree of the genus *Leishmania* is shown in **Fig 1**.

The current controversy is centred around the taxonomic status of the subclades of the Paraleishmania section. One problem is that it includes some species that were earlier classified as members of the genus *Endotrypanum*. However, there are serious concerns about the validity of this genus [25]. Some taxonomists revised and expanded this genus [19], whereas others treated it as nomen dubium and proposed a new section, Paraleishmania, essentially (although not formally) in its place [27].

The subgenus *L*. *(Viannia)* is restricted to the Neotropics, whereas the subgenus *L*. *(Leishmania)* occurs in both the New and Old World, referring to Middle and South America and southern Europe, Africa, and Asia, respectively. From the 53 currently recognized species [1], 29 are present in the Old World, 20 in the New World, three species in both, and only one species in Australia (*L*. *macropodum* [22, 28]). Moreover, 31 species are known to be parasites of mammals, and 20 species are pathogenic for humans, causing visceral leishmaniasis (VL), cutaneous leishmaniasis (CL), and mucocutaneous leishmaniasis [29, 30], affecting 1.2–2 million people annually (WHO report, 2016). The original grouping of species into subgenera was based on feature comparison such as the same parasite location in the vector’s intestine [31]. The phenotype-based system is further supported by the molecular analysis of, for example, the heat-shock protein 70 (HSP70) gene [32], the maxicircle-encoded ATPase subunit 6 gene [33], the cytochrome b (Cyt b) gene [34], the DNA/RNA polymerase gene sequences [35], or the 7SL RNA gene sequences [36]. Moreover, species within the subgenera can be grouped into complexes (based on hsp70 sequence differences) [32], although this is questioned by other studies [37].

The subgenus *L*. *(Sauroleishmania)* includes 21 species (such as *L*. *tarentolae*, *L*. *hoogstrali* [38], *L*. *adleri* [39], and *L*. *gymnodactyli* [40]), which are all restricted to the Old World and are transmitted by sand flies of the genus *Sergentomyia* [41, 42]. *Sauroleishmania* species primarily infect lizards [43]. However, *L*. *adleri* was also found to be able to infect mammals and has been associated with CL in humans [2, 44, 45].

There are three hypotheses for the origins of the genus *Leishmania*: the (1) Palearctic, (2) Neotropical, and (3) Multiple Origins hypotheses [24]. In the Palearctic hypothesis, the origin of *Leishmania* is assumed in Cretaceous lizards with recent migrations to the Nearctic and Neotropics, suggesting that *Sauroleishmania* forms a sister-clade to all other species [46–48]. In contrast, the second hypothesis suggests the origin of *Leishmania* in the Neotropics, supported by sequence-based phylogenies [25, 35, 49]. Here, data suggest that New World species emerged 46–34 million years ago and are the ancestors of Old World species [50], making *Sauroleishmania* a mammalian-derived and not an ancestral form. However, the Neotropical hypothesis requires two separate migrations to be valid: either a migration of the ancestors of *Leishmania*/*Sauroleishmania* to the Old World around 24–14 million years ago, followed by a second migration of a *Leishmania* subgenus back to the New World, or two migrations into the Old World by an ancestor of *Sauroleishmania* and, in addition, by an ancestor of an Old World *Leishmania* species [24, 50]. Last but not least, the Multiple Origin hypothesis suggests a division of *Leishmania* into the two lineages of Euleishmania (including the subgenera of *Leishmania*, *Viannia*, and *Sauroleishmania*) and Paraleishmania on the supercontinent Gondwana [25, 51]. Subsequently, the breakup of Gondwana separated the ancestors of all *Leishmania* subgenera. This is supported by recently published studies on large, multigene datasets (with some modifications) [24] and on the molecular evolution and phylogeny of *Leishmania* [52]. Based on current knowledge, the Multiple Origin hypothesis is the true hypothesis for the origin of the genus *Leishmania*. The phylogenetic position of *Sauroleishmania* between the subgenera *Leishmania* and *Viannia* also suggests that *Sauroleishmania* switched from mammalian to reptilian hosts [53–55].

### *L*. *tarentolae*—A model Leishmania system

The best-studied saurian-pathogenic species today is *L*. *tarentolae*, which was first isolated from the gecko, *Tarentola mauritanica*, in 1921 [5, 56]. The two most frequently used strain groups of *L*. *tarentolae* are the TAR strains, isolated by Parrot from an Algerian gecko in 1939 (e.g., TARII/UC and TARVI) [57, 58], and the LEM strains, isolated by Rioux from geckos in France (LEM-87, LEM-115, LEM-124, LEM-125, and LEM-306) [38]. *L*. *tarentolae* was also recently identified in reptile-biting *Sergentomyia minuta* sand flies in Spain [59]. Some of the old strains have been used for decades in basic research and cell culture because of the easy and safe handling. This would not be the case with human-pathogenic species. The commercially available strain P10 of Jena Bioscience is most likely based on the TARII/UC strain. In 2011, the genome of strain TARII/UC was sequenced [60], which has further confirmed the taxonomic status of *L*. *tarentolae* as a member of the genus *Leishmania* and increased the usefulness of this promising eukaryotic host for basic and applied research.

However, it was noted that prolonged cultivation of *L*. *tarentolae* without a selective pressure to maintain the ability to undergo a full life cycle resulted in genetic drift, which was remarkably noticeable in the mitochondrial genome. A recently published comparative study on the kinetoplast mitochondrial genome network (kDNA) of TARII/UC and LEM-125 shows that the TARII/UC strain has developed, unlike the LEM-125 strain, a partially defective mitochondrial genome [3]. It was further shown that the kDNA of *L*. *tarentolae* changes significantly during continuous culture from year to year [61], probably resulting in the loss of some proteins not required in cell culture [62]. Moreover, some LEM strains were shown to be transiently infectious [3, 4], which could raise the question of their current biosafety level.

The phenomenon of genetic drift is due to the high complexity of the kinetoplastid mitochondrial genetic system on the one hand and the lack of a mechanism that would ensure its orderly segregation in cell division on the other. In order to understand this lability of the kDNA, it is necessary to discuss the kinetoplast mitochondrial DNA and the replication and genetic role of the minicircles. This was intensively studied in *L*. *tarentolae*. The kDNA is a mass of DNA situated at the base of the flagellum [63, 64]. It consists of a species-dependent number (5,000–20,000) of small circular DNA molecules known as “minicircles,” all catenated together into a giant two-dimensional “network” which can be easily isolated because of its large size and stability [65, 66]. There is also a minor DNA species known as “maxicircles,” which is around 20–30 kb in size and also catenated to the network. The maxicircles are the equivalent of the mitochondrial DNA found in other eukaryotes and contain 18 conserved mitochondrial protein-coding genes, 12 of which are “cryptogenes” and contain encoded frameshifts or other defects that would prevent translation into functional proteins [67]. In the 1980s, it was discovered that transcripts of the mitochondrial cytochrome oxidase subunit II gene are modified posttranscriptionally by the insertion of four uridylate (U) residues at precise sites, which overcame a −1 frameshift and restored a translatable reading frame in the mRNA [68, 69]. This phenomenon was termed “RNA editing” and has therefore been studied in *L*. *tarentolae* and *Trypanosoma brucei* for more than 30 years. It was rapidly shown in other laboratories that the extent of editing varies extensively among the genes and the kinetoplastid species, with the most extreme cases requiring hundreds of editing events to generate a translatable mRNA out of a G- and A-rich skeletal sequence, a phenomenon which is known as “pan-editing.” For some time, the genetic role of the thousands of minicircles was not known, nor was the mechanism and the source of information that determined the precise number of inserted/deleted U residues into a preedited mRNA. In 1990, Blum and colleagues discovered in *L*. *tarentolae* that its kinetoplastid mitochondrion contains a novel class of small RNAs with the sequence information for the editing [70]. These RNAs were called “guide RNAs” (gRNAs) because they guide the enzymatic machinery to produce the precise insertion/deletion of U’s in the mRNA to make it complementary to the gRNA. At that time, an enzymatic cleavage/ligation model of editing was proposed, which has since proven essentially correct, although more complex than envisioned originally. It was initially found that gRNAs were encoded in the maxicircle DNA, but it soon was shown by using *L*. *tarentolae* that most were encoded in the minicircles, thus finally elucidating a biological role for the minicircles [71].

The number of gRNA genes and their location in the minicircles was found to vary among species. For example, *Leishmania* minicircles are around 900 bp and have a single gRNA gene located at a specific position, whereas *T*. *brucei* minicircles are around 1,000 bp and usually encode three gRNAs [72, 73]. Multiple gRNAs participate in editing of single pan-edited transcripts. The editing begins at the 3′-end of the mRNA and continues toward the 5′-end. The observed 3′–5′ polarity of editing is due to overlapping gRNAs, whereas each subsequent gRNA can extend the editing only after the preceding gRNA has completed its respective editing segment ("editing block") [74].

The thousands of catenated minicircles in a single kDNA network consist of multiple sequence “classes” depending on the specific gRNAs encoded. Each class is multicopy, but the actual number of copies per class fluctuates randomly. The rapid changes in frequencies of minicircle sequence classes were shown to be a direct consequence of the mode of replication and segregation of kDNA minicircles, which can be explained in terms of a model for kDNA minicircle DNA replication and segregation [66]. The model proposes that the kDNA network rotates during the S phase of the cell cycle. A type II topoisomerase randomly decatenates closed minicircles from the network, which replicate and then migrate to two antipodal nodes adjacent to the network where the daughter minicircles are recatenated onto the periphery of the network. Replicated minicircles are marked by the presence of nicks and gaps, and following replication of all network minicircles, covalent closure of all daughter molecules occurs in G2 phase. During cytokinesis, the doubled network splits into halves so that each daughter cell receives a kDNA network of the original size. However, recatenation of minicircles is essentially random, and therefore, the segregation of the minicircle classes during cell division is also random. This leads to stochastic variations in the minicircle copy numbers and, in extreme cases, may result in a complete loss of entire sequence classes. It is clear that a complete set of minicircle classes or gRNAs would be required for editing of all the cryptogene transcripts and translation. A loss of an entire minicircle class entails a loss of the respective gRNA and a disruption of editing. However, the cell can survive such a loss if the respective gene product is dispensable. It is not known if there are also rapid changes in the nuclear genomes.

The most dramatic example of this type of rapid genetic drift has occurred in two strains of *L*. *tarentolae*, as briefly mentioned above [3]. The old laboratory strain TARII/UC was shown to have lost a number of minicircle classes encoding specific gRNAs and thereby the translation of those genes, whereas the more recently isolated LEM-125 strain still contained all minicircle classes and gRNAs. It was hypothesized that these proteins were not required during life in culture. The loss of minicircles and gRNAs could also have an impact on recombinant protein expression. A rapid genetic drift in minicircle composition and sequence has also been observed in nature. *Trypanosoma cruzi* is responsible for Chagas disease in South and Central America. Morel and colleagues have observed that various *T*. *cruzi* strains exhibit different kDNA restriction digest profiles. It is now known that these differences were due to variation in the frequencies of minicircle sequence classes [75]. This has proved useful in studies of possible strain-dependent pathologies in humans. There are numerous other examples of rapid changes in minicircle heterogeneity that were used for discrimination and identification of other trypanosomatids, including strains and species of *Leishmania*.

### Life cycle and its geographical distribution

The life cycle of *Leishmania* species from the subgenera *L*. *(Leishmania)* and *L*. *(Viannia)* alternates between two main morphological forms: motile flagellated promastigotes in the insect (midgut and/or hindgut [76]) and nonmotile “rudimentary” flagellated amastigotes in specific immune cells (mostly macrophages) of the vertebrate host [77–79]. There is evidence of two separate, consecutive growth cycles during the development in the sand fly alone, consisting of four to five morphologically distinct life cycle stages: (1) procyclic promastigotes, (2) nectomonad promastigotes, (3) leptomonad promastigotes, and (4) metacyclic promastigotes [77, 80, 81]. Some studies also discuss another life cycle stage within the sand fly, the (5) haptomonad promastigotes, which represent a minor population and can be found attached to, e.g., the hindgut via the flagellar tip. However, their developmental origin is unknown, although likely to be derived from either leptomonad or nectomonad forms [77, 81]. Procyclic promastigotes divide in the midgut (within the blood meal) and subsequently become nondividing nectomonad promastigotes. This new form migrates to the anterior part of the gut and subsequently transforms into leptomonad promastigotes, which initiate the second growth cycle. *Leishmania* species of the subgenus *L*. *(Leishmania)* have in common that colonization of sand flies, *Phlebotomus* and *Lutzomyia*, is restricted to the midgut (suprapylarian) [31, 42]. Examples of well-known species are *L*. *donovani* (Old World) or *L*. *mexicana* (New World). Species of the subgenus *L*. *(Viannia)* colonize the midgut as well as the hindgut (peripylarian) of only *Lutzomyia* species. In a last step, leptomonads differentiate into nondividing metacyclic promastigotes (representing the mammalian-infective stage), which are transferred during a blood meal from the sand fly to the mammalian host [80]. Moreover, the leptomonads play a major role in the production of the promastigote secretory gel (PSG) plug. The PSG plug is needed to ensure successful transmission of *Leishmania* from the sand fly to its mammalian host, as the sand fly has to regurgitate the PSG plug in order to take a new blood meal [81, 82]. The PSG plug is transferred to the mammalian host together with metacyclic promastigotes. Inside the new host, metacyclic promastigotes ligate host macrophage receptors, which triggers phagocytosis and their internalization inside the parasitophorous vacuole of the macrophage [83, 84]. Subsequently, promastigotes transform into amastigotes, which multiply and infect other macrophages. Amastigote-infected macrophages can be taken up as part of the blood by a feeding sand fly, restarting the life cycle.

**Fig 2:**
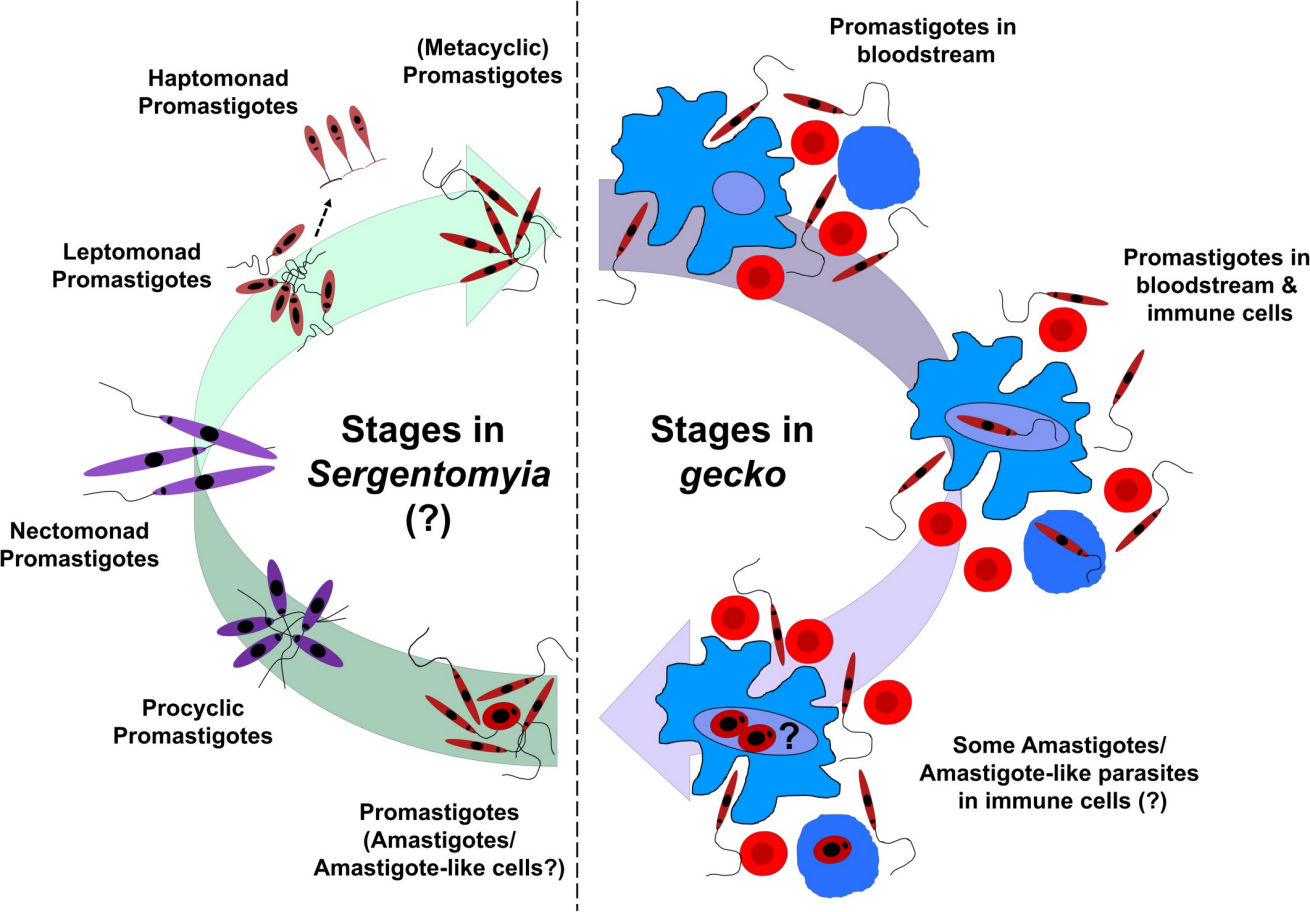
Simplified life cycle of *L*. *tarentolae* (*Sauroleishmania*). Sand fly (*Sergentomyia*) takes a blood meal from a gecko and thereby ingests promastigotes (major form) and some amastigotes/amastigote-like cells (minor form) of *L*. *tarentolae*. Inside the gut of the sand fly, immune cells burst, and the phagocytized part of Leishmania is released. Promastigotes colonize the lumen of the cloaca, the intestine (hindgut), and rarely, the blood of the sand fly. Although not known, it is possible that promastigotes (and amastigotes) of *L*. *tarentolae* undergo several transformations inside the sand fly, with stages of procyclic, nectomonad, leptomonad, haptomonad, and subsequently, metacyclic promastigotes (stages adapted from [80]). Metacyclic promastigotes would be the gecko-infective stage, and they are transferred back to a gecko during a new blood meal. Inside the gecko, promastigotes mainly live free in the blood. A small part is probably phagocytized by immune cells (monocytes and macrophages). Phagocytosed promastigotes might change back into amastigotes/amastigote-like cells, and the life cycle repeats. Immune cells are represented in blue, and erythrocytes are represented in red.

The complete life cycle of *Sauroleishmania* species is still debated. These parasites colonize the hindgut (hypopylarian) of *Sergentomyia* sand flies as promastigotes [31, 42], but some studies also claim colonization of the anterior midgut [85, 86]. In lizards (geckos), these parasites also dwell predominantly as promastigotes in the lumen of the cloaca and intestine, or in the bloodstream [87]. Amastigotes, either free or inside monocytes or erythrocytes, are seen only rarely [56, 85, 87, 88]. Transmission of *Sauroleishmania* to its vector has never been demonstrated, but it is believed to occur via a similar pool feeding mechanism, as described for mammalian vectors [80]. Compared with other sand fly genera, species of the genus *Sergentomyia* produce a relatively thick peritrophic matrix (the peritrophic matrix encloses the ingested blood meal), which might be one reason why the development of *Sauroleishmania* in the anterior midgut is not favored or possible [89, 90]. It is suggested that *Sauroleishmania* might be passed into the hindgut based on their inability to quickly escape from the peritrophic matrix [80]. Moreover, it is unknown whether metacyclic promastigotes are produced in *Sergentomyia* and whether there are additional developmental stages of *Sauroleishmania* (like nectomonads and leptomonads) inside the sand fly. However, the existence of a reptile-infecting promastigote form can be assumed. The proposed and simplified life cycle of *L*. *tarentolae* (*Sauroleishmania*) is summarized in **Fig 2**.

The geographical distribution of human-pathogenic *Leishmania* is closely linked to that of the insect vectors, which represent nearly 100 sand fly species of the genera *Phlebotomus* (42 species) in the Old World and *Lutzomyia* (56 species) in the New World [30]. These sand fly species inhabit tropical and subtropical zones of Asia, Africa, Australia, southern Europe, and the Americas between the latitudes 50° north and 40° south [30]. *L*. *tarentolae*–infected phlebotomine sand flies and geckos (*Tarentola annularis* and *T*. *mauritanica*) were found in southern Europe, North Africa, and the Middle East [5, 38, 41, 58, 91]. However, the prevalence of infection in gecko populations remains unknown. Moreover, the spread of *L*. *tarentolae* and other *Sauroleishmania* across its known geographical border might also be possible through climate change (allowing sand flies to reach new habitats) and because of the trade of hosts as pets.

### Similarities and differences to human-pathogenic species

From the more than 50 known *Leishmania* species, the genome from at least 15 has been fully sequenced, including the two sauroleishmanial species, *L*. *tarentolae* and *L*. *adleri* [2, 60, 92–102]. The data of most of these genomes can be found on the Kinetoplastid Genomics Resource database TriTrypDB (http://tritrypdb.org/tritrypdb/). Not many genomes of human-pathogenic *Leishmania* species were compared with the ones of *Sauroleishmania*. However, this would be highly advantageous, as it would give better insights into the evolution of the *Leishmania* genus and its split into the subgenera. Compared with the genomes of *L*. *major* (36 chromosomes, 32.8 Mb, 8,272 protein-coding genes) [92], *L*. *infantum* (36 chromosomes, 32.1 Mb, 8,154 protein-coding genes) [93], or *L*. *adleri* (38 chromosomes, 30.4 Mb, 7,849 protein-coding genes) [2], the genome of *L*. *tarentolae* (36 chromosomes, 31.6 Mb, 8,201 protein-coding genes) [60] contains similar attributes, and 90% of the gene content of *L*. *tarentolae* is shared with those human-pathogenic species. Hence, the important question is: What are the differences of the remaining approximately 10%? The analyses of the genome predict 95 coding sequences to be unique to *L*. *tarentolae* [60]. Furthermore, 250 genes found in human-pathogenic species were absent in the gecko parasite genome. Many of these 250 genes are preferentially expressed in the amastigote stage of the human-pathogenic species [60]. Although promastigotes of *L*. *tarentolae* are able to enter human phagocytic cells and differentiate into amastigote-like forms (the term “amastigote-like form” is explained in this section: Cell culture conditions and the phenotypes of *L*. *tarentolae*), there is no clear evidence for their replication within macrophages [4, 103, 104]. *L*. *tarentolae* does possess several virulence factors, such as cysteine protease B (CPB), lipophosphoglycan 3 (LPG3), and the leishmanolysin GP63 [105], but it lacks the major virulence factor amastigote-specific protein A2. A2 has been proven to play a major role in parasite virulence and visceralization capability [106]. Additionally, development of amastigotes inside lizards is still uncertain [87, 107]. Underrepresented genes and their products are normally involved in antioxidant defense or parts of the secretory pathway—e.g., vesicular-mediated protein transport. In detail, several adaptins are missing, which are involved in the formation of clathrin-associated adaptor protein complexes necessary for the formation of transport vesicles within the secretory pathway [108]. Other missing genes are glycosyltransferases (see genome of *L*. *major*, genes LmjF35.5250 and LmjF29.2110) or the endoplasmic reticulum chaperone calreticulin (LmjF31.2600), which ensures the folding and quality control of nascent secretory proteins [109]. In contrast, two gene families are enriched in *L*. *tarentolae* promastigotes—the previously mentioned leishmanolysin (GP63), a surface glyco-metalloprotease [110, 111], and promastigote surface antigen (PSA) proteins. Unlike other *Sauroleishmania* species, *L*. *adleri* has been shown to be able to infect mammals [2]. On the other hand, parasites of the *L*. *donovani* complex were shown to infect lizards [112]. Therefore, *Sauroleishmania* are not restricted to reptiles, and human-infecting *L*. *donovani* complex isolates can infect lizards and are likely transferred by *Sergentomyia* from human to lizard [112].

For *L*. *tarentolae*, in particular, the gene content differences could explain why they are not able to survive for many generations in human macrophages, why they are mostly found as free parasites in the insect vector, and why their amastigote form is still under debate. They seem to be better adapted to the promastigote and insect stages. However, the genome of the LEM strains should also be sequenced to uncover the differences between this strain, TARII, *L*. *adleri*, and the human-pathogenic species. Ideally, genomic DNA-sequencing platforms should use long-read technologies to effectively resolve centromeric and telomeric regions and to clarify whether a gene was not detected or lost due to assembly issues or read-length limitations. Suitable platforms are represented by, for example, PacBio or MiSeq [99].

## Biotechnological applications of *L*. *tarentolae*

**Fig 3:**
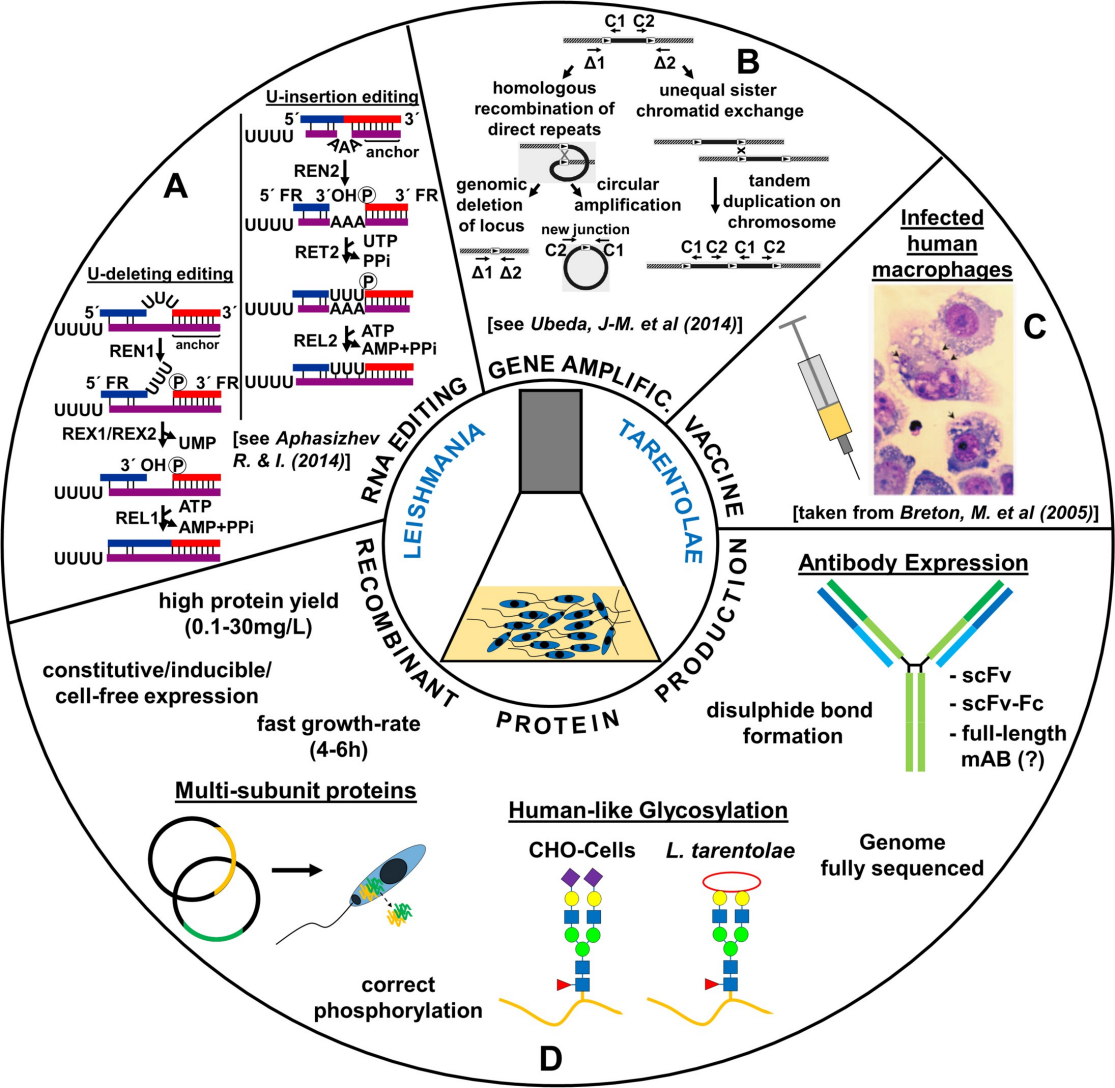
Biotechnological applications of the eukaryotic parasite *L*. *tarentolae*. *L*. *tarentolae* is used to study RNA editing (A), gene amplification (B), vaccine development against human-pathogenic *Leishmania* species (C), and recombinant (human) protein production (D). AMP, Adenosine monophosphate; ATP, Adenosine triphosphate; CHO, Chinese hamster ovary; PPi, diphosphate; REL, RNA editing ligase; REN, RNA editing endonuclease; RET, RNA editing TUTase; REX, RNA editing exonuclease; scFv, single-chain fragment variable; scFv-Fc, single-chain fragment variable fragment crystallizable; UMP, uracil monophosphate; UTP, uracil triphosphate. *Partial pictures adapted from [6] for (A), [118] for (B), and [103] for (C)*.

In the past 30 years, the parasite *L*. *tarentolae* has been utilized for a broad range of biotechnological and biomedical applications. In the beginning, the parasite was primarily used to study RNA editing [70, 113] and gene amplification [114, 115]. Later, applications were complemented by the use of the parasite as a vaccine candidate against human leishmaniasis in animal models [103, 104] and to screen for antileishmanial drugs [4]. Recombinant protein production is another important application area for several reasons [116, 117]. We will highlight the advantages of this eukaryotic protein expression system, describe available expression vectors, and discuss the cultivation conditions and its use as an immunotherapy agent. An overview of the biotechnological applications of *L*. *tarentolae* is shown in **Fig 3**.

### Advantages of the protein expression machinery of *L*. *tarentolae*

The expression machinery of *L*. *tarentolae* represents a competitive alternative to mammalian cell lines for the following reasons [119]: the parasite is probably not pathogenic to humans [5], it is relatively easy and cost effective to cultivate [10, 11], and it offers mammalian-like PTMs and satisfying recombinant protein yields averaging 0.1–30 mg/L [116]. Moreover, the expression system can easily be extended to an industrial production scale by growing the parasites in bioreactors and harvesting proteins in high throughput [11]. In a few examples, the protein yield could be increased to 500 mg/L (Jena Bioscience, LEXSY Conference presentation at Halle, Germany 2015), showing the general capacity of this parasite in culture. However, this is still around 10× less compared with many recombinant proteins expressed in mammalian cell lines such as Chinese hamster ovary (CHO cells; up to 5g/L) [120–122]. As it took around 25 years to increase the yield of proteins expressed in mammalian cell lines more than 100-fold to its 5 g/L nowadays [120], it can be expected that the yield of recombinant proteins expressed in *L*. *tarentolae* will reach a similar g/L range very soon. It is important to mention here that studies benchmarking the in vivo protein expression performance of the *L*. *tarentolae* system in direct comparison with other expression systems has never taken place. Therefore, its true success and average protein yields remain unknown to date. This lack of information could be one possible explanation why the *L*. *tarentolae* expression system has not further permeated the research and industrial sector.

Besides high protein yields, PTMs are also very important. In particular, protein glycosylation plays a major role in humans, as more than 50% of all human proteins are glycosylated [123], and besides proteins with enzymatic activity, glycoproteins (including monoclonal antibodies) are the most frequently approved recombinant therapeutic proteins [124, 125]. Moreover, many proteins need the (correct) glycosylation for their biological activity [17, 126, 127]. Glycosylation itself is a quality-control mechanism for the folding status of proteins [128]; it increases the half-life [129], and specific sugar epitopes can regulate the interaction with cell receptors.

To date, full-length antibodies have not been expressed in *L*. *tarentolae*. However, functional expression results of single-chain fragment variables (scFv’s) [12] and scFv-fragment crystallizable (scFv-Fc) fusion proteins [13] indicate that successful expression of full-length antibodies is only a matter of time [130]. The successful expression of multisubunit proteins, like the human heterotrimeric glycoprotein LM-322 [17,131], further underpins the general capacity of *L*. *tarentolae* to express full-length antibodies, as they also consist of several subunits. The potential application of *L*. *tarentolae* as an alternative platform for antibody expression has been recently reviewed [132].

Several other human glycoproteins were also successfully expressed in *L*. *tarentolae* [14, 116]. In particular, two human glycoproteins showed high posttranslational protein homogeneity to the human counterpart with complex biantennary N-glycans (EPO, [15]) and initial O-glycans (sAPPalpha, [16]). For EPO, only terminal sialic acids (N-acetylneuraminic acids) were missing. Sialic acids are terminal monosaccharides on many human glycoproteins [133] and are very important, as they directly regulate the immune response and influence the serum half-life of glycoproteins such as immunoglobulins (Ig) [127]. Current research indicates that enzymes for the sialic acid biosynthesis pathway are not present in any *Leishmania* species [134], but α2,3- and α2,6-linked sialic acids have been found on endogenous membrane glycoproteins of *L*. *major* and *L*. *donovani* [135–137]. To our knowledge, the mechanism behind the uptake of free sialic acid glycans by *Leishmania* is unknown. However, a CMP-sialic acid transporter (like the solute carrier family 35 transporter A1) in the Golgi apparatus membrane was assumed, transporting sialic acids from the cytoplasm into the Golgi [136, 137]. General uptake of free sialic acids is probably occurring through endocytosis. Adding the biosynthetic sialic acid pathway to *L*. *tarentolae* would be highly advantageous and increase the quality/similarity of expressed human recombinant proteins. This will be further discussed in the Discussion and outlook section.

Although not described for *L*. *tarentolae*, phosphorylation of the human recombinant cellular tumor protein p53 was successfully demonstrated in an avirulent strain of *L*. *donovani* [138], indicating that this phenomenon should also exist in *L*. *tarentolae* because of their close relationship. For all these reasons, *L*. *tarentolae* is a competitive alternative to mammalian cell lines.

### Available expression vectors

The expression system of *L*. *tarentolae* has been commercialized by Jena Bioscience and is known as LEXSY (http://www.jenabioscience.com/). Both constitutive [15] and inducible-integrative [10] expression vectors have been developed, which enable the production of intracellular or secretory recombinant proteins. By homologous recombination (double crossover), the expression cassette is either inserted into the chromosomal 18S ribosomal RNA locus (ssu; constitutive system) or into the ornithine decarboxylase locus (odc; inducible-integrative system) of *L*. *tarentolae*. ssu is a repetitive locus of the *L*. *tarentolae* genome with high rates of transcription by the host RNA polymerase I [15,139], whereas the odc locus is transcribed by RNA polymerase II. Gene expression regulation by RNA polymerase I is a unique attribute of *Leishmania* (including *L*. *tarentolae*) and probably all protozoan parasites of the order Kinetoplastida [140–142]. Furthermore, transcription in *Leishmania* is polycistronic: there are no introns (and therefore no *Cis*-splicing reactions), and posttranscriptional processing of pre-mRNA is effected by *Trans*-splicing reactions and polyadenylation within intergenic regions [143–146]. Protein expression regulation may be influenced by the structure of these intergenic regions [141,147].

Over the years, additional expression vector variants were developed, enabling protein expression in an inducible episomal [148] or cell-free system [149–151]. In the inducible systems, protein expression is activated through addition of the enhancer, tetracycline, being analogous to the well-known bacterial T7 RNA polymerase/tetracycline-controlled transcriptional activation (TET) repressor system [10]. It is expected that the T7-driven increase in mRNA production results in an increase in the recombinant protein yield. However, this advantage might be hypothetical, as the highly elevated mRNA production might be outstripped by the high degradation rates of exogenous transcripts. This has never been properly investigated but is important to validate the benefits of the *L*. *tarentolae* expression system for recombinant protein production. In the inducible episomal system, plasmids are maintained extrachromosomally as self-replicating episomes. Finally, proteins can be produced in *L*. *tarentolae* lysates using a unique cell-free approach in which the translation of endogenous mRNA is not suppressed by mRNA degradation but, instead, by antisplice leader oligonucleotides. This results in a much simpler system preparation compared with others. In general, species-independent translational sequences (SITSs) mediate cell-free protein synthesis, bypassing the early translation initiation factors. SITSs in combination with targeted suppression of endogenous mRNA of *L*. *tarentolae* are necessary factors to create a cell-free system. Recently, a large-scale benchmarking study showed that the cell-free system of *L*. *tarentolae* performs on par with the HeLa system (in terms of protein integrity and folding) [152]. This laid the foundation for further studies on, e.g., enzyme discovery and interactome analysis of caveolae [153,154]. Therefore, the cell-free system of *L*. *tarentolae* has the potential of becoming a widespread and important tool in applications like enzyme discovery, metabolic engineering, or gene circuit prototyping in the context of eukaryotic proteome analysis.

For functional validation, enhanced green fluorescent protein (eGFP) has been expressed in all expression vector variants. Furthermore, constitutively expressed proteins—for example, the Ca^2+^-dependent serine protease proprotein convertase 4 (PC4) [155], the chloramphenicol acetyl transferase (CAT) [156], and the successfully crystallized Cu/Zn superoxide dismutase (SOD1) [157]—were also expressed in the inducible episomal system [148]. Also, several human Rab GTPases (guanosine triphosphate hydrolysing enzyme) tagged with eGFP were expressed in the inducible-episomal [148] as well as in the cell-free system [149,150]. Recently, the crystal structure of the channelrhodopsin 2 protein could be solved when expressed in *L*. *tarentolae* using the integrative inducible expression system [158]. More expressed proteins of different origins and expression vector variants can be found in the review by Basile and colleagues from 2009 [116] and in the article by Kushnir and colleagues from 2011 [148].

### Cell culture conditions and the phenotypes of *L*. *tarentolae*

**Fig 4:**
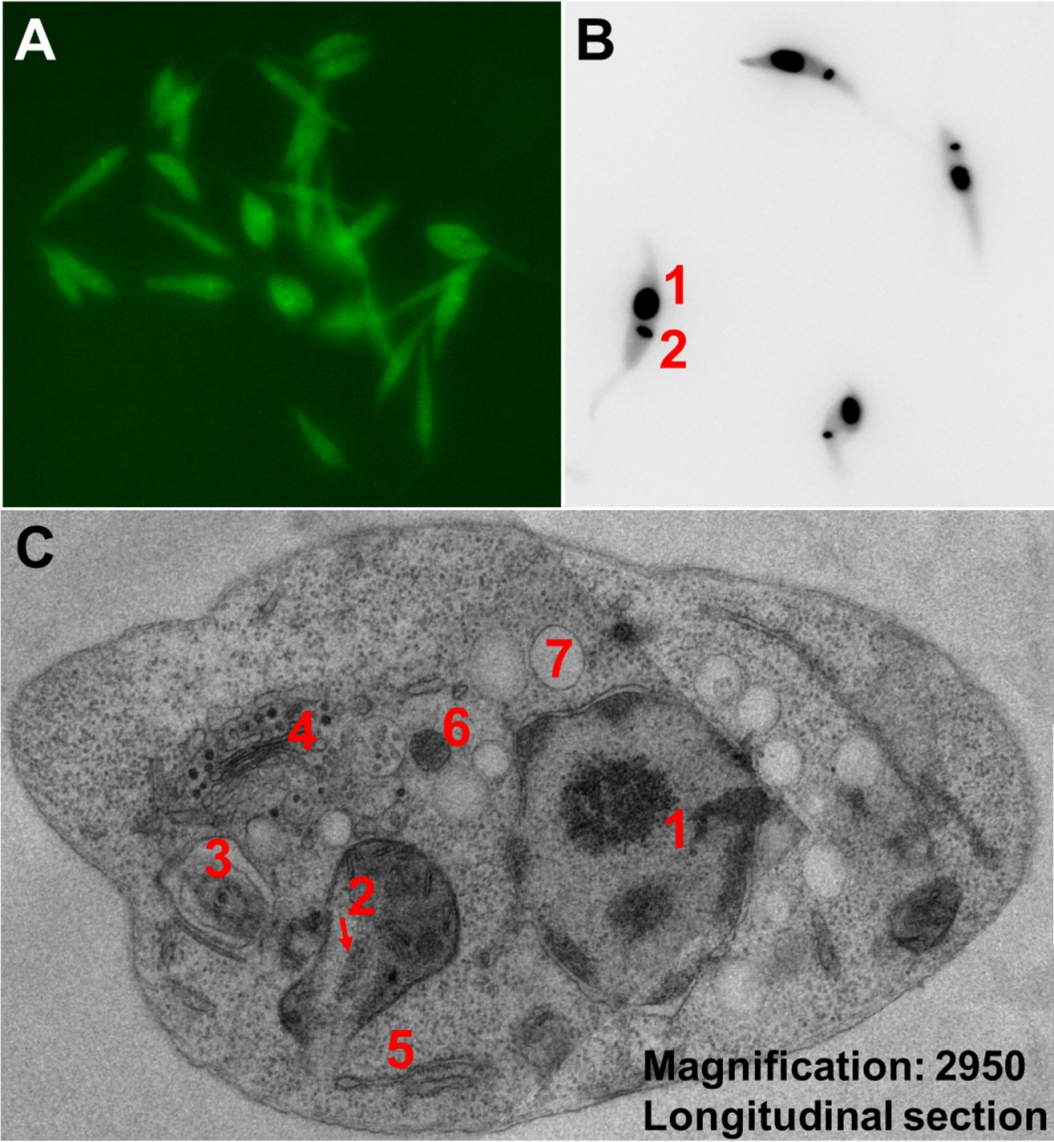
Microscopy images of *L*. *tarentolae*. (A) Fluorescent *L*. *tarentolae* promastigotes expressing eGFP. (B) *L*. *tarentolae* promastigotes stained with DAPI, highlighting the nucleus (1) and the kDNA (2). (C) TEM (two fused images) of an *L*. *tarentolae* promastigote, longitudinal cell section, with a magnification of ×2,950. Cell nucleus (1), kinetoplast inside single mitochondrion (2), flagellum within flagellar pocket (3), Golgi apparatus (4), rough endoplasmic reticulum (5), glycosome (6), and acidocalcisome (7). eGFP, enhanced green fluorescent protein; kDNA, kinetoplastid DNA; TEM, transmission electron microscopy.

In cell culture, the parasite is found in its motile promastigote form. Promastigotes are monoflagellated and of lance-like structure with a length of 4–12 μm and a width of 0.5–3 μm (**Fig 4A and 4B**). Amastigotes as well as amastigote-like cells (and even dividing promastigotes) are smaller and of round-like shape with a rudimentary flagellum.

In general, promastigotes divide every 4–6 hours by single fission—their growth rate is dependent on the oxygen supply. They are cultivated in agitated suspension culture under aerobic conditions at 25–27°C, which is the optimal growth temperature in the sand fly host. In contrast, the body temperature in geckos is quite variable [159]. There is no need of expensive CO_2_ incubators or sera. For growth, different complex and synthetic media are available for selection [160]. Hemin, an iron-containing porphyrin, is essential for promastigote growth and survival [161]. Depending on the selected growth medium, the cell concentration can reach a maximum of up to 400 million cells/ml under optimal growth conditions. Clonal selection can be normally performed on agar plates after 5–10 days postinoculation by using one or more out of the six available selection markers/antibiotics (nourseothricin, blasticidin, bleomycin, Geniticin G418, hygromycin, and puromycin).

The occurrence of *L*. *tarentolae* amastigotes is still under debate. Past experiments tried to trigger the differentiation of promastigotes to amastigotes from human-pathogenic *Leishmania* species by mimicking phagolysosomal conditions [162,163]. In detail, the temperature was raised to 32 or 37°C, the pH was acidified to 5.5 or 4.5, and in some cases, parasites were additionally cultivated in a CO_2_ incubator with 5%–7% CO_2_. Analyses of typically expressed amastigote proteins and gene expression profile studies revealed slight differences between isolated and induced amastigotes [164,165]. Based on those differences, induced amastigotes are referred to as the “amastigote-like” form today [165]. Induction of amastigote-like forms of *L*. *tarentolae* has also been successfully performed for strain LEM-125 but not for the sequenced strain, TARII/UC [4]. Like other *Leishmania* species, *L*. *tarentolae* offers some unique organelles, like glycosomes [166–168] and acidocalcisomes [169], and a specific secretory pathway [170]. A transmission electron-microscopy image of these organelles of *L*. *tarentolae* can be found in **Fig 4C**.

### *L*. *tarentolae* as an immunotherapy agent

There are numerous drugs available to treat human leishmaniasis [171]. However, because of the enormous side effects of many drugs combined with their high costs, the requirement of long hospitalization, and the increasing emergence of drug-resistant strains, chemotherapy treatment of leishmaniasis has become a challenging approach [172]. In addition, there is no vaccine available that provides long-term protection and immunity against leishmaniasis [171]. In recent years, recombinant proteins were expressed in nonpathogenic *L*. *tarentolae* for the purpose of improving vaccine potential. In one example, a recombinant *L*. *tarentolae* strain was generated expressing the sand fly protein *Phlebotomus papatasi*–secreted salivary protein 15 (PpSP15), a salivary antigen. Vaccination with *L*. *tarentolae*-PpSP15 in combination with deoxynucleotides of cytosine triphosphate and guanine triphosphate linked via a phosphodiester (CpG) as a prime-boost modality conferred strong protection against *L*. *major* infection in BALB/c mice because of a high production of interferon gamma (IFN-ɣ) and interleukin 17 (IL-17) [173]. In another study, they used *L*. *tarentolae* as a live vaccine expressing the *L*. *donovani* A2 antigen along with two cysteine proteinases as a trifusion gene. Here, the recombinant *L*. *tarentolae* strain protected BALB/c mice against *L*. *infantum* infection [18]. Moreover, *L*. *tarentolae* harboring cysteine proteinase and A2 genes has been successfully tested as a prophylactic vaccine in a dog model against *L*. *infantum* [174]. However, the largest drawback of plasmid DNA vaccine candidates usually is its inefficient intracellular delivery, resulting in low levels of gene expression, which in turn limits the immune response [175]. Therefore, the long-term effectiveness of live vaccines and the host immune response against *Leishmania* infections must be carefully evaluated. In two other studies with the goal to improve the treatment of CL, they used transgenic *L*. *tarentolae* expressing human neutrophil peptide-1, IFN-ɣ, or IFN-ɣ-induced protein 10 (CXCL-10), respectively. Recombinant proteins were used as a prophylactic vaccine as well as a therapeutic tool against *L*. *major*–infected BALB/c mice, resulting in improved treatment potential of CL [176,177]. These examples show the great potential of *L*. *tarentolae* as an immunotherapy agent.

## Useful online tools/databases

A few online tools/databases are available that can facilitate the work with *L*. *tarentolae* and expand personal knowledge. On the one hand, TriTrypDB (www.tritrypdb.org) can be used as a genomic database to search for specific genes or to find out the occurrence of genes between different *Leishmania* species [178]. In detail, TriTrypDB is an integrated database offering the datasets of several *Leishmania* and *Trypanosoma* species. Another next generation–sequencing database is Leish-ESP (http://leish-esp.cbm.uam.es/). It contains transcriptomic and genomic data of several *Leishmania* species, including *L*. *braziliensis* [101]. On the other hand, LeishCyc can be used to study biochemical pathways (metabolomics) of *Leishmania* species using datasets of *L*. *major* [179,180]. LeishCyc is a useful guide to build up metabolic pathways and for metabolic data visualization. The U insertion/U deletion Edited Sequence Database (http://164.67.82.180/trypanosome/database.html) contains sequences of mitochondrial genes and cryptogenes from kinetoplastid protozoa. Edited sequences and translated amino acid sequences are also provided. A novel "map" format provides the edited RNA sequence aligned with the genomic DNA sequence and the translated amino acid sequence; both U deletions and U insertions are indicated by gaps in the edited sequence or the genomic sequence.

## Discussion and outlook

*L*. *tarentolae* has been extensively studied for more than 30 years, with the conducted research having covered a broad set of areas. For example, it has become a model organism to study kDNA organization and RNA editing [67, 70,181], was genetically modified numerous times to produce human recombinant proteins for biotechnological and biomedical research [116], and is becoming a promising therapeutic tool to enable treatment against human leishmaniasis [176,177], just to name a few. In this review, which is focused on the biotechnological aspects, we tried to cover only the relevant application areas. We think that human recombinant protein expression in *L*. *tarentolae* has the potential to become a recognized alternative to mammalian cell lines, if the following issues are addressed accordingly: (1) Humanization of the *L*. *tarentolae* glycoprotein expression system; (2) Detailed analysis of the T7-driven increase in mRNA production; (3) Recombinant protein expression in different *L*. *tarentolae* strains or phenotypes. The potential suitability of the *L*. *tarentolae* expression system for human recombinant full-length antibodies has been reviewed elsewhere [132]. (4) Moreover, we recommend that the proteomes of *Sauroleishmania* species be analyzed and the genome of other species of this subgenus (in addition to *L*. *tarentolae* [60] and *L*. *adleri* [2]) be fully sequenced, as this would help to further understand the development of the genus *Leishmania* and its separation into the subgenera. We will further outline each of the four mentioned points below.

### Humanization of glycoproteins expressed in *L*. *tarentolae*

In general, glycoproteins play a major role, as more than 50% of all human proteins are glycosylated [123]. Moreover, they are the most frequently approved recombinant therapeutic proteins [124, 125]. To increase the homogeneity of recombinant glycoproteins (glycan pattern) expressed in *L*. *tarentolae*, different glycosyltransferases can be transfected. Here, sialic acids are the most important, as these terminal sugar residues directly regulate the immune response and directly influence the serum half-life of glycoproteins, e.g., Ig [127]. *Leishmania* lack enzymes for the sialic acid biosynthesis pathway [134], although terminal sialic acids (α2,3- and α2,6-linked) were found on endogenous membrane glycoproteins of *L*. *major* and *L*. *donovani* [135–137]. To humanize the glycosylation profile of *L*. *tarentolae*, the TS gene can be integrated to express the TS protein, as recently suggested [182]. The TS is an enzyme of *Trypanosoma* species that can transfer sialic acids to endogenous glycoproteins [183,184]. Successful expression of the TS and its functionality, verified by the proof of sialylated recombinant proteins, could already be demonstrated with insect cells [185] and yeasts [186]. Additionally, it was also successfully expressed in *L*. *major*. Here, the enzyme was used to investigate the influence on the virulence, not for recombinant protein expression [135]. TS expression in *L*. *tarentolae* would enable the production of human-like, sialylated glycoproteins (like full-length antibodies) with a comparably low work load and much impact for many biomedical applications.

### T7-driven increase in mRNA production

It is expected that the T7-driven increase in mRNA production results in an increase in the recombinant protein yield. However, this advantage might be a hypothetical one, as the highly elevated mRNA production might be outstripped by the high degradation rates of exogenous transcripts. Therefore, we suggest investigating this issue in the near future, as it is important to validate the benefits of the *L*. *tarentolae* expression system for recombinant protein production.

### Recombinant protein expression in different *L*. *tarentolae* strains or phenotypes

To date, the vast majority of all *L*. *tarentolae*–based research has been performed on the TARII/UC strain, which has a partially defective mitochondrial genome [3]. It is unknown whether this defect might have a negative impact on recombinant protein expression. The mitochondrial genome of strain LEM-125 is still intact [3]. We suggest testing the ability of LEM-125 to produce recombinant proteins, either in promastigotes or in amastigotes/amastigote-like cells. The cell-free protein expression system could also be applied here. Our rationale to use amastigotes/amastigote-like cells is the following: promastigotes are adapted to a life as extracellular motile cells in the gut of insects, whereas amastigotes are adapted to survive the intracellular conditions of immune cells of vertebrate hosts [187]. For instance, promastigote survival depends on the adhesion of parasitic surface gycosylphosphatidylinositol (GPI) proteins to receptors on the cells lining the intestinal wall of the insect vector [188]. In contrast, strong reduction of surface GPI proteins in amastigotes [170,189] is an important adaptation process to survive in vertebrates, resulting in decreased immune response [137]. Hence, without differentiation, amastigotes would not be able to establish infections in macrophages, and promastigotes would not be able to survive/reproduce in the gut of insects. In cell culture, *Leishmania* are often maintained as promastigotes. Mimicking phagolysosomal conditions can induce amastigote-like cells of *L*. *tarentolae* [162,163]. However, induction of amastigote-like cells was only successful with *L*. *tarentolae* strain LEM-125 and not for the sequenced strain TARII/UC [4]. The question is: Would amastigotes or amastigote-like cells be more suitable for human recombinant protein expression because of their evolutionarily acquired adaptations to the vertebrate host? It is known that, during differentiation, the PTM of endogenous proteins changes [190]. This could also be the case for recombinant proteins, resulting in a higher homogeneity of the PTM to the mammalian counterpart. This raises the question of whether the LEM-125 strain would be a better recombinant protein expression alternative. However, it is mostly unknown whether the change of the phenotype has an influence on the translation of the introduced gene, protein expression, and modification. So far, only green fluorescent protein (GFP) was episomally expressed in amastigote-like cells, but without recombinant protein analysis [106], showing that despite morphological change, the gene was still transcribed and led to a functional product. Alternatively, an avirulent designed Leishmania strain of, for example, *L*. *major*, could be used to produce human recombinant proteins. In summary, it needs to be investigated in the future whether amastigote-like cells of *L*. *tarentolae* strain LEM-125 or amastigotes/amastigote-like cells of human-pathogenic species are better suited (higher homogeneity of PTMs to the original counterpart) for human recombinant protein expression than promastigotes.

### Genomic analyses of more *Sauroleishmania* species

There are 21 known species of the subgenus *Sauroleishmania* [1], and most of them have not been fully sequenced so far. They represent an evolutionary product of switching from mammalian to reptilian hosts. Based on the transient infection capabilities of *L*. *adleri* and *L*. *tarentolae* (LEM-125) [2, 4], one could assume that both species might have retained a relic capability of infecting mammals, or it is a contaminative/nonspecific infection. This question can be addressed by a comparable genomic analysis involving additional sauroleishmanial and human-pathogenic species, or by using infection models with mice or human cell cultures.

When the previously suggested issues are all successfully applied, it can be expected that the *L*. *tarentolae* expression system will be an adequate alternative and work on par with mammalian cell lines and therefore be much more attractive for applied science in the medical sector.

**Key learning points**

1. The *Sauroleishmania* subgenus includes 21 species of sand fly–transmitted parasites, which primarily infect reptiles (lizards) as vertebrate hosts. Molecular phylogenetic data unequivocally support the evolutionary origin of *Sauroleishmania* from parasites of mammals.
2. The two most frequently used *L*. *tarentolae* strains are the TAR strains [TARII/UC, from which the P10 strain from Jena Bioscience was most likely derived, and TARVI] and the LEM strains (LEM-87, LEM-115, LEM-124, LEM-125, and LEM-306). Strain LEM-125 was shown to transiently infect mammals, including humans, which could raise the question of its biosafety level, making future studies on strain-related differences absolutely necessary.
3. Recombinant protein production in *L*. *tarentolae* is a promising application area because of its ease and low cost of cultivation, the high yields of recombinant proteins, and a homogenous human-like glycosylation pattern.
4. It is anticipated that successful full-length antibody expression may be achieved within a few years, either in promastigotes of *L*. *tarentolae* strain TARII or in amastigote-like cells of strain LEM-125, or in an avirulent strain of a human-pathogenic *Leishmania* species. Humanization of the glycosylation profile can be achieved with, for example, the transfer of TS gene.
5. Recombinant *L*. *tarentolae* can be used for the generation of prophylactic vaccines or for the development of a therapeutic approach against human leishmaniasis. This has been successfully shown in a mouse model.

**Top five papers**

1. Akhoundi M, Kuhls K, Cannet A, Votýpka J, Marty P, Delaunay P, Sereno D. A Historical Overview of the Classification, Evolution, and Dispersion of Leishmania Parasites and Sandflies. PLoS Negl Trop Dis. 2016 Mar 3;10(3):e0004349. 10.1371/journal.pntd.0004349. eCollection 2016 Mar.
2. Didwania N, Shadab M, Sabur A, Ali N. Alternative to Chemotherapy-The Unmet Demand against Leishmaniasis. Front Immunol. 2017 Dec 21;8:1779. 10.3389/fimmu.2017.01779. eCollection 2017.
3. Coughlan S, Mulhair P, Sanders M, Schonian G, Cotton JA, Downing T. The genome of Leishmania adleri from a mammalian host highlights chromosome fission in Sauroleishmania. Sci Rep. 2017 Mar 3;7:43747. 10.1038/srep43747.
4. Simpson L, Douglass SM, Lake JA, Pellegrini M, Li F. Comparison of the Mitochondrial Genomes and Steady State Transcriptomes of Two Strains of the Trypanosomatid Parasite, Leishmania tarentolae. PLoS Negl Trop Dis. 2015 Jul 23;9(7):e0003841. 10.1371/journal.pntd.0003841. eCollection 2015.
5. Raymond F, Boisvert S, Roy G, Ritt JF, Légaré D, Isnard A, Stanke M, Olivier M, Tremblay MJ, Papadopoulou B, Ouellette M, Corbeil J. Genome sequencing of the lizard parasite Leishmania tarentolae reveals loss of genes associated to the intracellular stage of human pathogenic species. Nucleic Acids Res. 2012 Feb;40(3):1131–47. 10.1093/nar/gkr834. Epub 2011 Oct 13.
